# Cross protective efficacy of the Non-Neurotropic live attenuated herpes simplex virus type 1 vaccine VC-2 is enhanced by intradermal vaccination and deletion of glycoprotein G

**DOI:** 10.1016/j.vaccine.2022.09.015

**Published:** 2022-09-14

**Authors:** Brent A. Stanfield, Fernando J. Bravo, David A. Dixon, Vladimir N. Chouljenko, Konstantin G. Kousoulas, David I. Bernstein

**Affiliations:** a Division of Biotechnology and Molecular Medicine and Department of Pathobiological Sciences, School of Veterinary Medicine, Louisiana State University, Baton Rouge, LA, USA; b Cincinnati Children’s Hospital Medical Center, University of Cincinnati, Cincinnati, OH, USA

**Keywords:** Human Herpes Simplex Vaccine, Guinea Pig model, Genital Herpes, Intradermal vaccination, VC-2, Glycoprotein G

## Abstract

Herpes simplex virus type 1 and 2 (HSV-1 and HSV-2 respectively) cause life-long latent infections resulting in recurrent orofacial and genital blisters or sores. Ensued disease can be painful and may lead to significant mental anguish of infected individuals. Currently, there are no FDA-approved vaccines for either prophylactic or therapeutic use, and recent clinical trials of subunit vaccines failed to achieve endpoints goals. Development of a safe live-attenuated herpes simplex vaccine may provide the antigenic breadth to ultimately protect individuals from acquiring HSV disease. We have previously shown that prophylactic use of the non-neurotropic live attenuated HSV-1 vaccine, VC-2, provides potent and durable protection from genital HSV-2 disease in the guinea pig model. Here, we investigated the effects of intradermal administration as well as the deletion of the viral glycoprotein G (gG) on the efficacy of prophylactic vaccination. Vaccination with either VC-2, VC-2 gG null, or gD2 MPL/Alum offered robust protection from acute disease regardless of route of vaccination. However, both the VC-2 gG-null and the ID vaccination route were more effective compared to the parent VC2 administered by the IM route. Specifically, the VC-2 gG-null administered ID, reduced HSV-2 vaginal replication on day 2 and day 4 as well as mean recurrent lesion scores more effectively than VC2 administered IM. Most importantly, only VC-2 gG null IM and VC-2 ID significantly reduced the frequency of recurrent shedding, the most likely source for virus transmission. Similarly, while all vaccinated groups demonstrated a significant reduction in the number of animals testing PCR-positive for HSV-2 in their dorsal root ganglia following challenge only VC2 ID vaccinated animals demonstrated a significant reduction in DRG viral load. All vaccinations induced neutralizing antibodies to HSV-2 MS when compared to unvaccinated guinea pigs. Therefore, further investigation of VC-2 gG null delivered ID is warranted.

## 1. Introduction

Human herpes simplex virus type 1 and 2 (HSV-1 and HSV-2 respectively) are alpha herpes viruses that infect the peripheral nervous system. They are highly transmittable and successful pathogens that infect a large portion of the adult human population globally. Specifically, in the United States new HSV-2 infections are expected to grow by more than 600,000 cases per year [1]. Genital HSV-2 infection begins in the epithelium and may produce acute disease manifesting as blisters and genital lesions primarily driven by the immunopathogenesis of the host in response to infection. Following the resolution of acute disease, the hallmark of all alpha herpes viruses is the “latency” phase of infection. This describes how the virus resides in the neuronal ganglia of the host in a transcriptionally repressed state leading to life-long infection. During periods of immune suppression, the virus will reactivate either asymptomatically or symptomatically with the afflicted individual shedding infectious virus or additionally developing recurrent disease [Reviewed in [2,3]. HSV infection increases the risk of acquiring HIV [4-6], can cause life-threatening encephalitis [7] and may also contribute to the development of currently irreversible age-related neurological disorders like Alzheimer’s disease [8,9]. The development of a prophylactic HSV vaccine is a priority [2,10,11].

There is no approved vaccine for HSV. The recent Herpevac clinical trial using a gD2 subunit vaccine initially showed promise in HSV seronegative women [12] but failed in larger clinical trials to protect against HSV-2 infection or disease [13]. A need for further refinement of existing technologies to carry forward into clinical trials exists. We have previously demonstrated the protective efficacy of the non-neurotropic VC2 vaccine [14] and tested the long term (greater than6 months) durability of this response with success [15]. Vaccination with VC2 also demonstrated long term neutralizing antibody responses (greater than250 days following vaccination) in rhesus macaques [16]. Here, using the guinea pig model of genital HSV-2 disease, we explored two approaches for improving its protective efficacy by deletion of gG, (VC2 gG Null) or altering the route of vaccination [from intramuscular (IM) to intradermal (ID) vaccination, VC2 Parental ID]. The gG deletion was initially constructed to allow detection of wild type HSV-1 or HSV-2 infection post vaccination using commercially available kits that identify seroconversion to gG1 or gG2.

## 2. Materials and methods

### 2.1. Vaccines and adjuvants

As previously detailed {Stanfield, 2014 #183}, the VC2 non-neurotrophic live-attenuated herpes simplex vaccine (VC2 Parental), was constructed by the KG Kousoulas laboratory (Louisiana State University, Baton Rouge, Louisiana) utilizing the two step double-Red recombination protocol implemented on the cloned HSV-1(F) genome in a bacterial artificial chromosome (BAC) plasmid [16,17]. VC2 contains the gKΔ31-68 deletion (37 aa; gK aa 31–68) in the amino terminus of gK as well as a deletion of the amino-terminal 19 amino acids (UL20 aa 4–22). The VC2 gG Null was also constructed by deletion of the gG (US4) open reading frame by the Kousoulas laboratory (Louisiana State University, Baton Rouge, Louisiana) utilizing the two-step double-red recombination protocol implemented on the cloned VC2 Parental genome in a bacterial artificial chromosome (BAC) plasmid. Targeted mutagenesis of gK, UL20, and gG were confirmed by next generation whole genome sequencing. No other nucleotide changes were detected comparing the parental HSV-1(F) BAC, VC2 Parental, and VC2 gG Null. The vaccines were diluted with DMEM for intramuscular (IM) or intradermal (ID) administration.

The gD2 vaccine was prepared by G. Cohen (University of Pennsylvania) from Sf9 (Spodoptera frugiperda) cells (GIBCO BRL) infected with a recombinant baculovirus expressing gD2 as previously described [18,19]. The MPL/Alum combination contained 50 μg of MPL (Sigma-Aldrich Corporation, St. Louis, MO) and 200 μg of Alhydrogel (2%) (Accurate Chemical and Scientific Corporation, Westbury, NY) [18]. The gD2 + MPL/Alum vaccine was prepared by mixing 5 μg of purified gD2 with 100 μL of MPL/Alum adjuvant.

### 2.2. Viruses and cells

HSV-2 strain MS (ATCC-VR540) was grown to high titer and quantified by plaque assay in primary rabbit kidney cells as previously described [20]. VC2 Parental and VC2 gG Null were grown and titered on Vero cells as previously described [17].

### 2.3. Animals

Female Hartley guinea pigs (251–350 g) were purchased from Charles River Laboratories (Wilmington, MA) and housed under AAALAC approved conditions at Cincinnati Children’s Hospital Medical Center (CCHMC). All procedures and protocols were approved by the CCHMC Institutional Animal Care and Use Committee.

### 2.4. Study design and methods

To evaluate protection from HSV-2 challenge, 5 groups of 12 female Hartley guinea pigs (250–300 g, 60 animals total) were vaccinated and challenged with virulent HSV-2 (MS strain): group 1: no vaccine control, group 2: 1 × 10^6^ plaque forming units (pfu) VC2 Parental intramuscular (IM) into the quadriceps, group 3: 1 × 10^6^ pfu VC2 gG Null (IM), group 4: 1 × 10^6^ pfu VC2 Parental intradermal (ID) injected into the skin proximal to the lumbar spine, and group 5: 5 μg gD2 + MPL/Alum (IM). A total volume of 100 μL per vaccination was used. ID injection was performed by shaving and sanitizing the site of injection. A 27-gauge syringe was used at an angle of approximately 10° to insert the needle bevel side up approximately 3 mm into the dermis. The full 100 μL was injected resulting in a small blister indicating ID injection. Animals were vaccinated 3 times at 63, 42, and 21 days prior to challenge. Animals were then challenged with HSV-2 MS strain intravaginally by first using a pre-moistened (sterile saline) calcium alginate swab (Puritan Calgiswab Type 3 Guilford, ME) to disrupt the vaginal membrane of each animal. 1 × 10^6^ pfu of HSV-2 was then instilled into the vaginal vault in a 0.1 ml suspension using a 1 ml slip tip syringe [21].

Vaginal swabs were collected on select days and titers determined on Vero cells to determine acute vaginal virus replication. A lesion score-scale was used to measure the occurrence to symptomatic disease during acute and recurrent phases of infection ranging from 0 representing no disease to 4 representing severe vesiculoulcerative skin disease of the vulva and perineum [22]. The acute and recurrent lesions were scored daily and presented as a cumulative daily score of the means for each animal over time. During the recurrent phase of infection animals were vaginally swabbed three times a week from day 21–62 to assess recurrent shedding. We acknowledge that the selection of day 21 as the starting point for recurrent shedding is somewhat arbitrary however in our experience with this model and the nature of adaptive immune responses 21 days post infection is optimal to begin measuring the change from acute virus infection to reactivation events. Total DNA was extracted from the vaginal swabs followed by quantitative polymerase chain reaction (qPCR) analysis using primers specific to HSV-2 gG (see below) to determine the frequency of viral shedding [23]. The guinea pigs were sacrificed for necropsy at the end of the study, and the spines were harvested and frozen (−80 °C) dorsal root ganglia (DRG) were collected from the stored tissue and total DNA extracted to quantify the latent viral burden by HSV-2 specific qPCR (see below).

### 2.5. qPCR of HSV-2 DNA

To assess HSV-2 shedding during recurrent disease and the latent viral load of HSV-2 in DRGs, HSV-2 gG2 specific qPCR was performed. The gG2 specific primers and probe sequences used are previously described (23) and were as follows:

Forward: 5′-CGG/AGA/CAT/TCG/AGT/ACC/AGA/TC-3′; reverse: 5′-GCC/CAC/CTC/TAC/CCA/CAA/CA-3′; and probe: 5′-FAM-ACC/CAC/GTG/CAG/CTC/GCC/G-tamRA-3′.

Each 20 μL reaction contained 100 ng of total DNA (collected from vaginal swabs or isolated from excised DRG), 0.5 μM of each forward and reverse primer, 0.10 μM of probe, and 10 μL of 2x Taq-man Master Mix (ABI). PCR amplification was performed on a 7500 Fast Real-Time PCR system (ABI). Thermocycling consisted of a pre-incubation step at 50 °C for 2 min and an initial denaturation step 95 °C for 10 min. This was followed by 40 cycles of first a denaturation step at 95 °C for 15 s, followed by an annealing step at 60 °C for 1 min, and an elongation step at 72 °C for 10 s. To precisely quantify the genomic abundance of HSV-2 a standard curve was generated with ten-fold serial dilutions of purified HSV-2 DNA (ATCC) containing from 100,000 to 1 HSV-2 copies suspended in 50 ng of DNA extracted from uninfected guinea pig brain. The limit of detection of this qPCR assay was determined to be a minimum of 5 genome copies, with excellent linearity (R ≥ 0.98) over 5 logs of HSV genomic DNA as previously described (23).

### 2.6. Measurement of neutralizing antibody activity

Serum samples collected 21 days after the third vaccination (prior to challenge) were heat inactivated at 56 °C for 30 min. Heat inactivated serum was then serially diluted in two-fold increments from 1:4 to 1:2048 in DMEM containing 10% rabbit complement (Cedarlane, Burlington, NC) and mixed with a fixed amount of HSV-2 (~100 pfu) and incubated for 1 h at 37 °C as previously described [24]. The virus/complement/serum mixtures were then applied to plates containing Vero cells and incubated at 37 °C for 1 h to allow for viral attachment/entry. Following incubation, a 1.5% methylcellulose overlay was applied to the cells to promote plaque formation. After 3 days of culture at 37 °C, the cells were fixed and staining with Crystal Violet (Sigma-Aldrich, St. Louis, MO). Plaques were counted after visualization on a dissecting microscope. The dilution producing a ≥ 50% reduction in plaques was considered the neutralizing antibody endpoint for each sample and presented as LOG_10_ geometric mean titer (GMT).

### 2.7. Statistical analysis

Student’s *t* test was performed using two-tailed analysis for comparison between two groups. ANOVA was initially performed for all comparisons between multiple groups larger than 2 followed by Tukey’s post-hoc test. Data are presented as means of the sample group and bars represent standard deviations about the mean. Incidence data or parts of a whole data were compared by Fishers’ exact test. A P value < 0.05 was considered significant.

## 3. Results

### 3.1. VC2 vaccine efficacy of acute disease

**Fig. 1.**
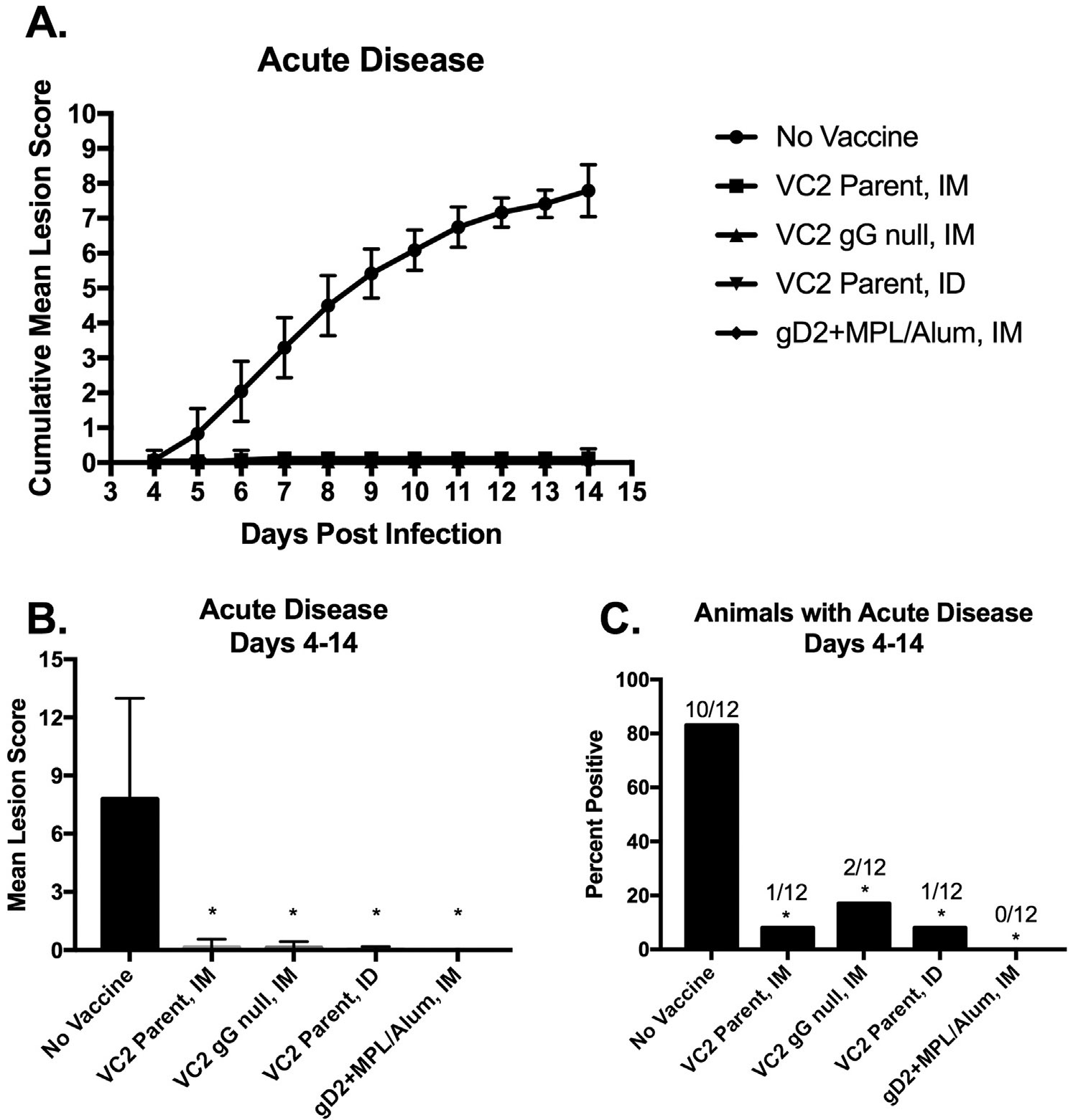
Pathogenesis of Acute Disease Days 4–14. **A.** Cumulative Mean Lesion Score of guinea pigs left unvaccinated or vaccinated with either VC2 Parent (IM), VC2 gG Null (IM), VC2 Parent (ID), or gD2 + MPL/Alum (IM) and challenged intravaginally with 1 × 10^6^ PFU of HSV-2 (MS Strain). Daily scores were collected on a scale from 0 to 4 and the average added cumulatively over the period. Error bars represent standard deviation of the mean. **B.** Comparison of cumulative mean lesion scores at the end of the acute disease period from day 4 to 14 post challenge. **C.** The percent of animals that developed acute genital disease over the acute disease period. The number of animals with disease over the number without disease is presented above each bar. * P < 0.05 vs. No Vaccine.

Guinea pigs were either left unvaccinated or vaccinated with either VC2 Parental Intramuscularly (IM), VC2 gG Null IM, VC2 Parental Intradermally (ID), or gD2 + MPL/Alum IM. As demonstrated in Fig. 1 all vaccinated animals were significantly protected from acute disease following vagina HSV-2 MS challenge. Only unvaccinated animals developed significant disease over the 10-day observation period (Days 4–14) (Fig. 1A and 1B) and all vaccination approaches significantly reduced the occurrence of vaginal disease over this period (Fig. 1C).

**Fig. 2.**
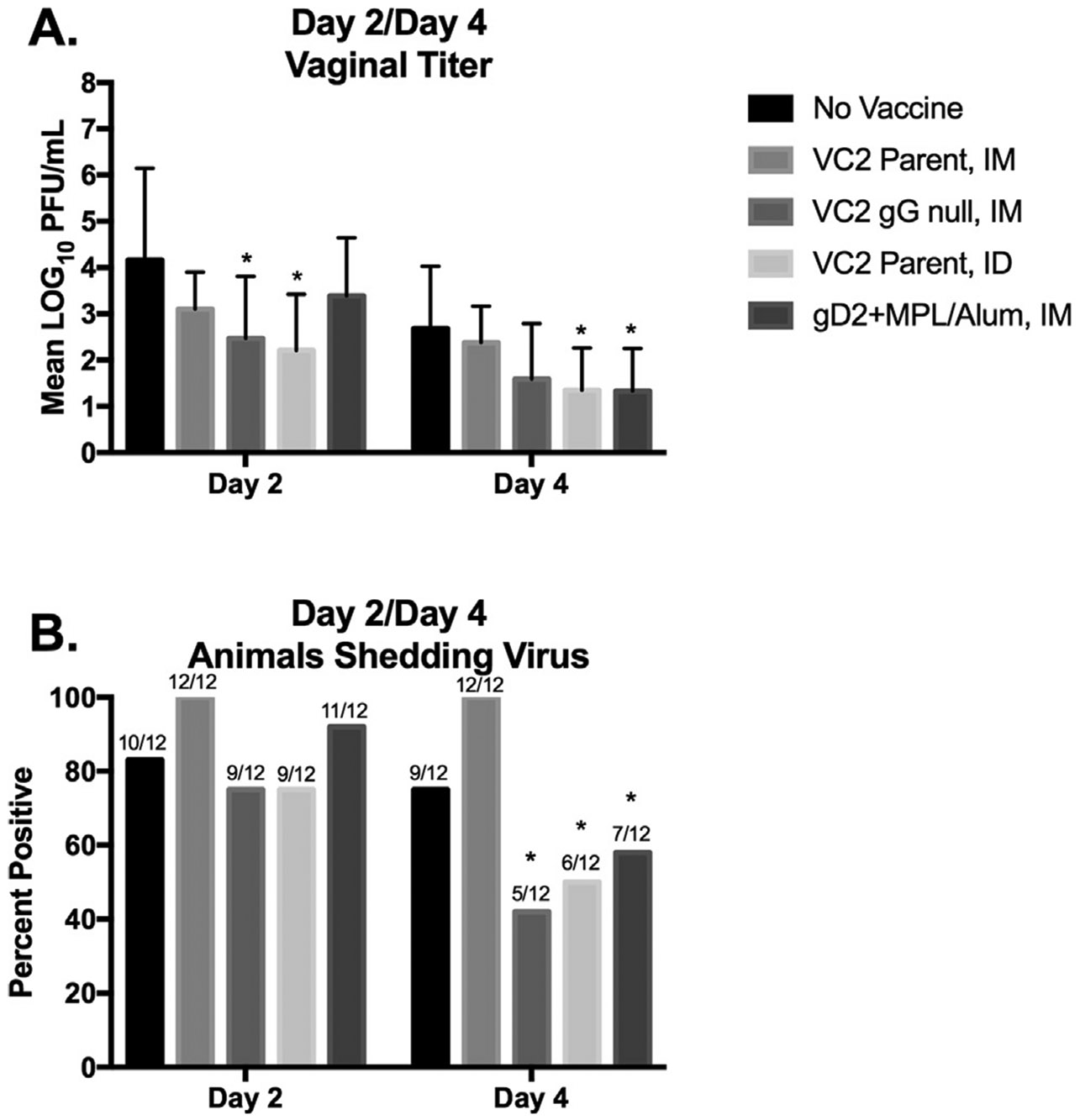
Vaginal Shedding at days 2 and 4 post challenge. **A.** Mean LOG_10_ PFU/mL was quantified from vaginal swabs collected at days 2 and 4 post challenge of guinea pigs left unvaccinated or vaccinated with either VC2 Parent (IM), VC2 gG Null (IM), VC2 Parent (ID), or gD2 + MPL/Alum (IM) and challenged intravaginally with 1 × 10^6^ PFU of HSV-2 (MS Strain) by plaque assay. **B.** The percentage of animals with swabs testing positive for infectious HSV-2 on days 2 and 4 post challenge. The number of animals testing positive over the total number of animals per group is presented above each bar. * P < 0.05 vs. No Vaccine.

Reduction of virus replication is another and often more sensitive way to compare vaccines. Interestingly, only vaccination with VC2 gG Null IM or VC2 Parental ID demonstrated a significant reduction in vaginal viral titers at Day 2 post infection (Fig. 2A). VC2 Parental ID continued to demonstrate a significantly reduced vaginal shedding on Day 4 post infection as did gD2 + MPL Alum IM (Fig. 2A). Despite not reaching reduced viral shedding at Day 4 post infection, the VC2 gG Null IM demonstrated a significantly reduced number of animals with detectible virus (5/12) on Day 4 post infection (Fig. 2B). VC2 Parental ID and gD2 + MPL Alum IM also significantly reduced the frequency of animals testing positive at Day 4 post infection (6/12 and 7/12 respectively) (Fig. 2B).

**Fig. 3.**
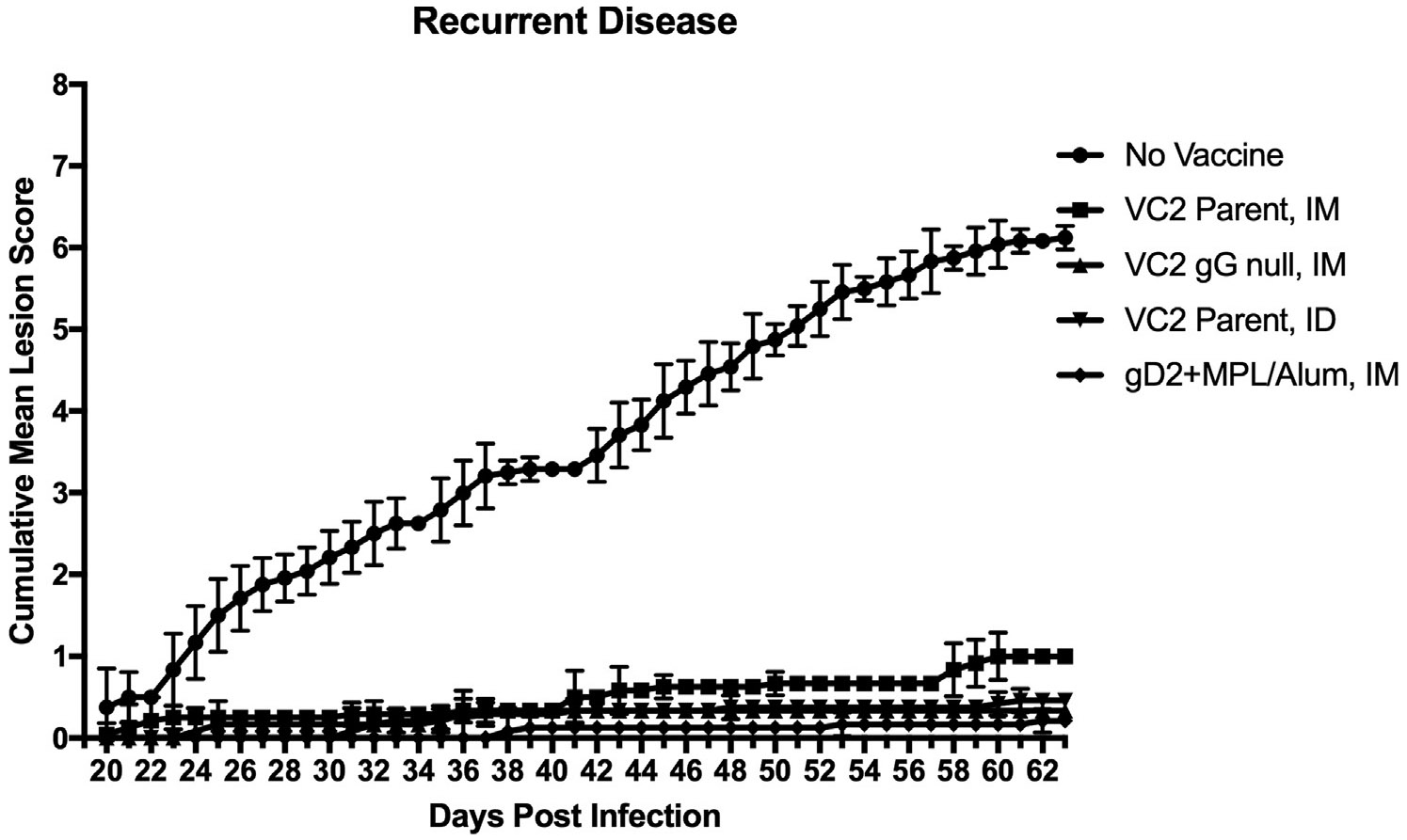
Cumulative Mean Lesion Score of Recurrent Disease. Cumulative Mean Lesion Score of recurrent HSV-2 disease in guinea pigs left unvaccinated or vaccinated with either VC2 Parent (IM), VC2 gG Null (IM), VC2 Parent (ID), or gD2 + MPL/Alum (IM) and challenged intravaginally with 1 × 10^6^ PFU of HSV-2 (MS Strain). Daily scores were collected on a scale from 0 to 4 and the average added cumulatively over the period.from day 20–63 Error bars represent standard deviation of the mean.

**Fig. 4.**
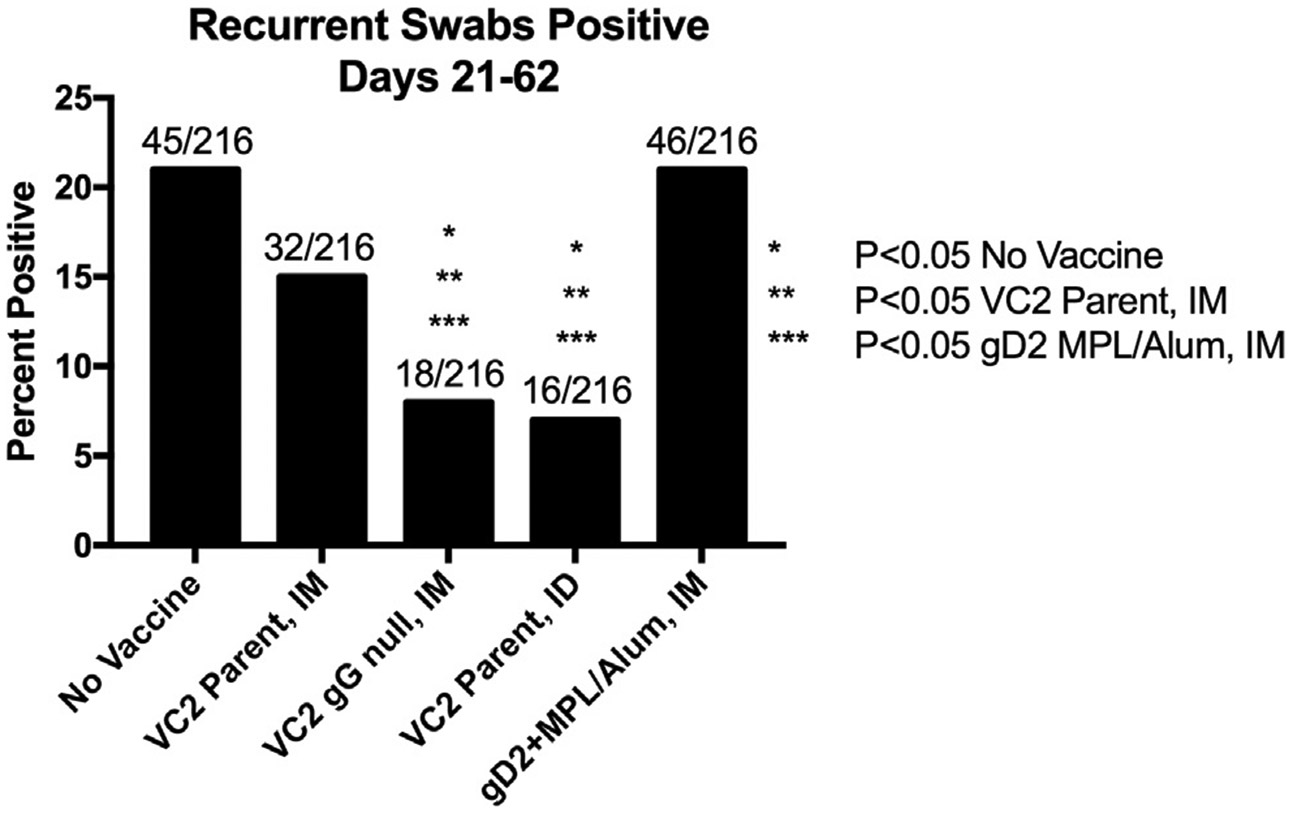
Viral Shedding During Period of Recurrences. The percentage of vaginal swabs testing PCR positive for HSV-2 DNA over the recurrent phase of disease. The number of swabs testing positive over the total number of swabs per group is presented above each bar. * P < 0.05 No Vaccine, ** P < 0.05 VC2 Parent (IM), *** P < 0.05 gD2 MPL/Alum (IM).

Recurrent disease was monitored from Days 20–63 post infection. Most of the disease (Cumulative Mean Lesion Score) developed in the unvaccinated animals (Fig. 3). During the recurrent phase of disease guinea pigs were swabbed 3 times per week for the detection of shed virus by quantitative PCR. This amounted to a total of 216 swabs per treatment group (1080 swabs total). From this analysis no significant differences in animals with detectible viral shedding between groups was detected, as most animals shed on at least one day (10/12–12/12). Similarly mean LOG_10_ Genome Copies/μg of DNA (~100 copies per positive sample) were equivalent. (Data not shown). However, the number of recurrent swabs testing PCR positive during this period were significantly (p < 0.05) reduced in the VC2 gG Null IM and VC2 Parental ID vaccinated groups (18/216 and 16/216 respectively) when compared to No Vaccine, VC2 Parent IM, and gD2 + MPL Alum IM groups (45/216, 32/216, and 46/216 respectively) (Fig. 4).

**Fig. 5.**
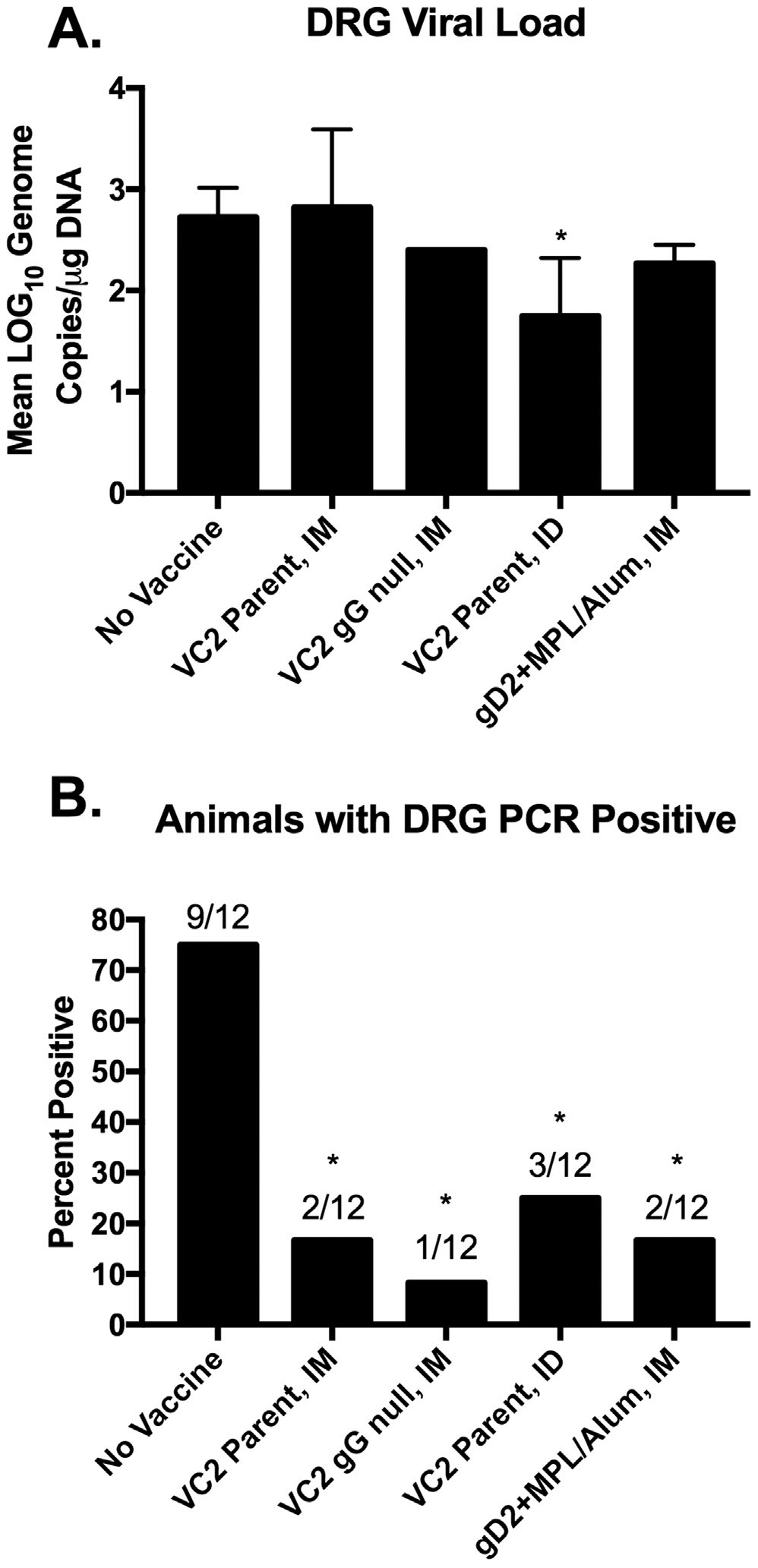
Neuroprotection from latent HSV-2 Infection by Vaccination. **A.** Mean LOG_10_ genome copies/μg of DNA was quantified by qPCR from total DNA isolated from sacral dorsal root ganglia collected at the end of the study. **B.** The percentage of animals with sacral dorsal root ganglia testing PCR positive for HSV-2 DNA at the end of the study. The number of animals testing positive over the total number of animals per group is presented above each bar. * P < 0.05 vs. No Vaccine.

Following the recurrent phase of infection guinea pigs were sacrificed at day 63 post infection. Doral root ganglia (DRG) were collected and processed for total DNA extraction for quantification of neuronal viral load. Only the VC2 Parent ID vaccinated group demonstrated a significant reduction in detectable DRG viral load in animals testing PCR + of HSV-2 DNA in their DRG (Fig. 5A). However, all vaccinated groups demonstrated a significant reduction in the frequency of animals testing positive for DRG HSV-2 genomes (Fig. 5B).

**Fig. 6.**
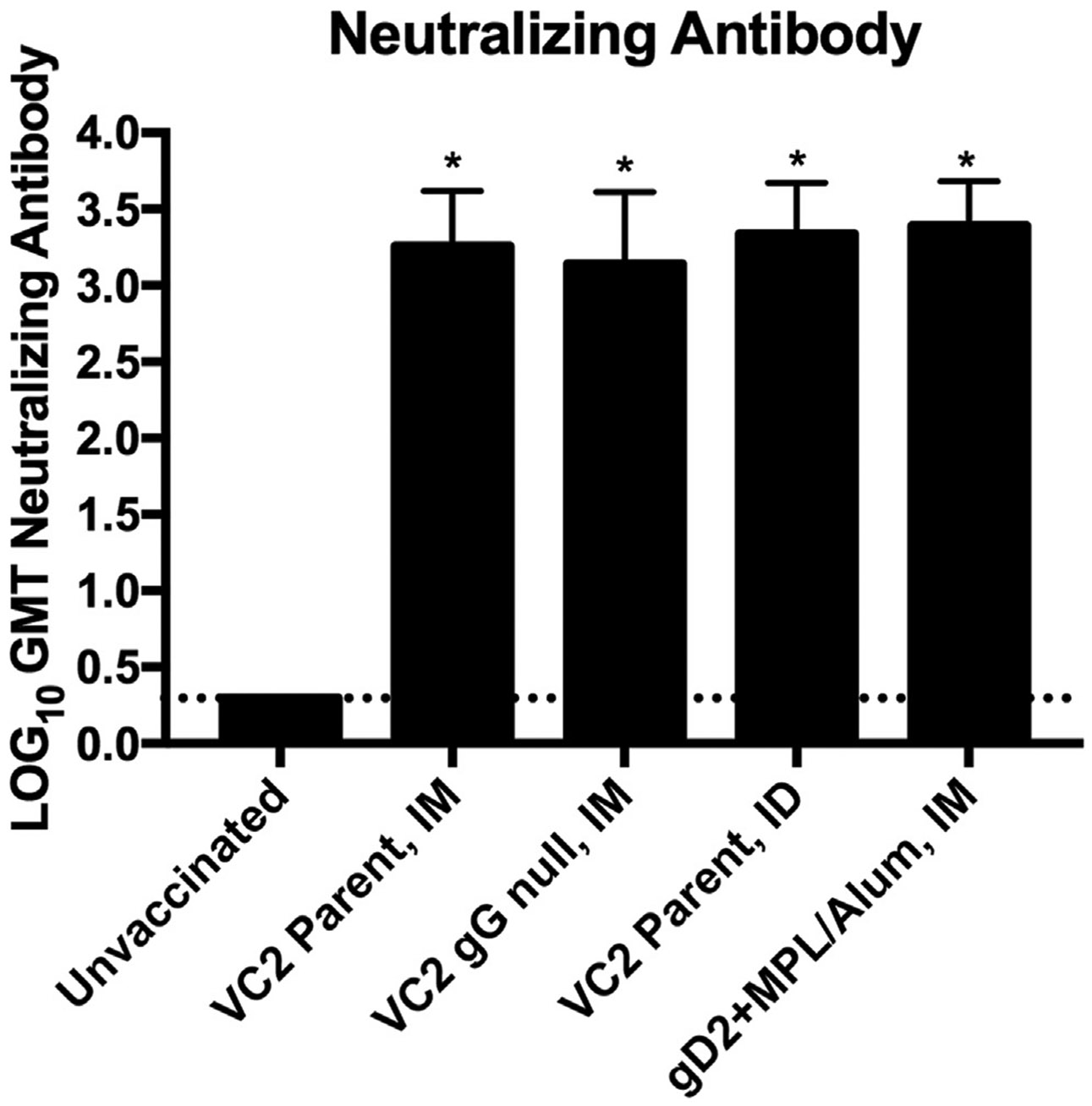
Neutralizing Antibodies Generated by Vaccination. LOG_10_ geometric mean titer (GMT) Neutralizing Antibody was calculated for serum obtained from guinea pigs after vaccination but before challenge with HSV-2 MS. The endpoint was defined as the highest dilution of serum showing a 50% or greater reduction in viral plaques compared to the HSV-2/complement control. * P < 0.05 vs. No vaccine.

All vaccinated animals demonstrated a significant increase in HSV-2 specific neutralizing antibodies (p < 0.0001). Loss of gG in VC2 gG null did not enhance the cross-neutralizing efficacy of the VC2 vaccine (Fig. 6).

## 4. Discussion

The development of HSV vaccines has been delayed with the failure of the adjuvanted gD2 vaccine [13] and the halt of the HSV15 study (ClinicalTrials.gov Identifier: NCT04222985). However, there are many promising technologies poised to begin clinical trials in the coming years [3)] Live attenuated vaccines offer the opportunity to present many more of the immunogenic HSV proteins than the gD and gB vaccines previously tested [25]. Previous success of the live-attenuated neurotropic varicella zoster virus (VZV) vaccine (Zostavax) suggest that a live-attenuated herpes simplex vaccine may provide significantly enhanced protection over subunit vaccines from HSV infections and resultant immunopathogenesis [2] Ideally, given the safety profile of subunit and mRNA vaccines, prophylactic and/or therapeutic vaccines will transition away from live-attenuated once we gain an understanding of a successful vaccine approach against HSV in humans.

We have previously tested the non-neurotropic VC2 vaccine’s ability to protect guinea pigs from acute HSV-2 disease in prophylactic, therapeutic, and durability of protection studies [14,15]. Results from these studies have demonstrated the vaccine to be safe and effective and provided protection from acute disease as well as reducing the frequency of recurrent shedding when compared to gD2 + MPL/Alum [14]. These promising results indicate that further development of the VC2 vaccination strategy is warranted to improve vaccine’s efficacy. Here, we report that deletion of the viral glycoprotein G (VC2 gG Null) or altering the route of vaccination from intramuscular to intradermal vaccination significantly enhance the neuroprotective phenotype of the VC2 vaccine. However, we acknowledge the difference in the protection demonstrated by the parental VC2 vaccine delivered via IM injection compared to our previously published results [14,15]. Specifically, in our previous studies, VC2 delivered via IM vaccination demonstrated a significant reduction in vaginal shedding at both day 2 and 4 post challenge. Here, VC2 IM did not perform significantly better than unvaccinated animals. Additionally, in this study all VC2 IM vaccinated animals had detectable viral shedding during the acute phase of disease while our previous work demonstrated only 4/12 animals having detectable viral shedding. We attribute this to the variability of the outbred guinea pig model of genital HSV-2 disease and the relatively small numbers of animals used in these studies. It is for these reasons we include previously tested vaccination strategies (VC2 parental IM and gD2 + MPL/Alum) as controls in our investigation.

Evaluation of prophylactic HSV vaccines should include protection from infection as well as disease. For subunit vaccines it is relatively easy to determine if there has been a subclinical infection, as one can measure seroconversion to non-vaccine HSV antigens to distinguish infection from vaccination. For live attenuated vaccines it is more difficult, as there may be no dominant HSV antigens that are not present in the vaccine virus. Glycoprotein G (gG) is a non-essential component of the viral membrane and is the most anti-genically divergent cell surface antigen between HSV-1 and HSV-2 with no cross-reactive immunogenic regions [31]. We therefore deleted gG because it is the prototypic antigen for serodiagnosis of either HSV-1 or HSV-2 infection [26-30]. Thus, we developed the VC2 gG Null vaccine strain: as a live-attenuated vaccine suitable for large scale clinical trials and monitoring of type specific seroconversion. VC2 gG Null replicates to titers comparable to the VC2 Parental *in-vitro*, allowing for the simple production of high-titer virus stocks (Chouljenko and Kousoulas, Unpublished).

Surprisingly, deletion of gG, VC2 gG Null IM appeared to provide improved protection compared to the parent VC2 when administered IM. VC2 gG Null IM significantly reduced vaginal viral titers at 2 days post infection as well as reducing the number of animals shedding infectious virus by 4 days post infection (Fig. 2). Importantly, vaccination with VC2 gG-Null IM also significantly reduced the frequency of recurrent shedding, the most frequent mode of HSV-2 transmission, compared to unvaccinated, VC2 Parental IM, and gD2 + MPL/Alum IM (Fig. 4). Perhaps because of a significant reduction in DRG viral load detected in this group. The reason for the improved protection is currently being examined.

We have previously reported that vaccination with another live attenuated vaccine, R2, was more effective when administered ID compared to intramuscular (IM) and intravaginal (IVag) administration (23). Intradermal vaccination has also demonstrated increased efficacy of the VC2 vector expressing tumor neo-antigens (VC2-Ova) in a syngenic melanoma model by almost completely eliminating tumor cell proliferation and metastasis [32]. Here we report that protection from neuronal infection by HSV-2 was enhanced by ID vaccination with the VC2 vaccine (VC2 Parental ID). Like VC2 gG-Null IM, VC2 Parental ID demonstrated a significant reduction in the frequency of viral reactivation during recurrent disease (Fig. 4). This correlates with a significant (10-Fold) reduction in DRG viral load in VC2 Parental ID vaccinated animals at day 63 post infection (Fig. 5). Both VC2 gG Null IM and VC2 Parental ID performed equally well protecting animals from recurrent disease. The enhanced protection offered by VC2 Parental ID confirms that ID vaccination enhances live-attenuated HSV-1 vaccine protection from heterologous HSV-2 challenge in a guinea pig model of acute and recurrent genital disease. Obviously, the next step is to combine the more protective approaches identified in this trial, ID route of vaccination and VC2 gG Null.

Here we demonstrate that the intradermal vaccination provides enhanced VC2 induced protection in comparison to intramuscular vaccination and that deletion of gG from VC2 increases cross protective immunity to pathogenic HSV-2 challenge in the guinea pig model of cute and recurrent disease. VC2 gG Null vaccination may be further enhanced by ID vaccination, which will be tested in future studies. Additional prophylactic and therapeutic studies are warranted.
